# Functional Characterization of Clinical Isolates of the Opportunistic Fungal Pathogen Aspergillus nidulans

**DOI:** 10.1128/mSphere.00153-20

**Published:** 2020-04-08

**Authors:** Rafael Wesley Bastos, Clara Valero, Lilian Pereira Silva, Taylor Schoen, Milton Drott, Verônica Brauer, Rafael Silva-Rocha, Abigail Lind, Jacob L. Steenwyk, Antonis Rokas, Fernando Rodrigues, Agustin Resendiz-Sharpe, Katrien Lagrou, Marina Marcet-Houben, Toni Gabaldón, Erin McDonnell, Ian Reid, Adrian Tsang, Berl R. Oakley, Flávio Vieira Loures, Fausto Almeida, Anna Huttenlocher, Nancy P. Keller, Laure Nicolas Annick Ries, Gustavo H. Goldman

**Affiliations:** a Faculdade de Ciências Farmacêuticas de Ribeirão Preto, Universidade de São Paulo, Ribeirão Preto, Brazil; b Department of Medical Microbiology and Immunology, University of Wisconsin—Madison, Madison, Wisconsin, USA; c Department of Pediatrics, University of Wisconsin—Madison, Madison, Wisconsin, USA; d Faculdade de Medicina de Ribeirão Preto, Universidade de São Paulo, Ribeirão Preto, Brazil; e Department of Biomedical Informatics, Vanderbilt University School of Medicine, Nashville, Tennessee, USA; f Life and Health Sciences Research Institute, School of Medicine, University of Minho, Braga, Portugal; g Life and Health Sciences Research Institute/3B’s Associate Laboratory, Guimarães, Portugal; h Laboratory of Clinical Bacteriology and Mycology, Department of Microbiology, Immunology and Transplantation, KU Leuven, Leuven, Belgium; i National Reference Center for Mycosis, University Hospitals Leuven, Leuven, Belgium; j Centre for Genomic Regulation, Barcelona, Spain; k Life Sciences Program, Barcelona Supercomputing Centre, Barcelona, Spain; l Mechanisms of Disease Program, Institute for Research in Biomedicine, Barcelona, Spain; m ICREA, Barcelona, Spain; n Centre for Structural and Functional Genomics, Concordia University, Montreal, Quebec, Canada; o Department of Molecular Biosciences, University of Kansas, Lawrence, Kansas, USA; p Instituto de Ciência e Tecnologia, Universidade Federal de São Paulo, São José dos Campos, Brazil; q Department of Biological Sciences, Vanderbilt University School of Medicine, Nashville, Tennessee, USA; University of Georgia

**Keywords:** *Aspergillus nidulans*, clinical isolates, genome sequencing, metabolomics

## Abstract

Aspergillus nidulans is an opportunistic fungal pathogen in patients with immunodeficiency, and virulence of A. nidulans isolates has mainly been studied in the context of chronic granulomatous disease (CGD), with characterization of clinical isolates obtained from non-CGD patients remaining elusive. This study therefore carried out a detailed biological characterization of two A. nidulans clinical isolates (CIs), obtained from a patient with breast carcinoma and pneumonia and from a patient with cystic fibrosis that underwent lung transplantation, and compared them to the reference, nonclinical FGSC A4 strain. Both CIs presented increased growth in comparison to that of the reference strain in the presence of physiologically relevant carbon sources. Metabolomic analyses showed that the three strains are metabolically very different from each other in these carbon sources. Furthermore, the CIs were highly susceptible to cell wall-perturbing agents but not to other physiologically relevant stresses. Genome analyses identified several frameshift variants in genes encoding cell wall integrity (CWI) signaling components. Significant differences in CWI signaling were confirmed by Western blotting among the three strains. *In vivo* virulence studies using several different models revealed that strain MO80069 had significantly higher virulence in hosts with impaired neutrophil function than the other strains. In summary, this study presents detailed biological characterization of two A. nidulans *sensu stricto* clinical isolates. Just as in Aspergillus fumigatus, strain heterogeneity exists in A. nidulans clinical strains that can define virulence traits. Further studies are required to fully characterize A. nidulans strain-specific virulence traits and pathogenicity.

**IMPORTANCE** Immunocompromised patients are susceptible to infections with opportunistic filamentous fungi from the genus *Aspergillus*. Although A. fumigatus is the main etiological agent of *Aspergillus* species-related infections, other species, such as A. nidulans, are prevalent in a condition-specific manner. A. nidulans is a predominant infective agent in patients suffering from chronic granulomatous disease (CGD). A. nidulans isolates have mainly been studied in the context of CGD although infection with A. nidulans also occurs in non-CGD patients. This study carried out a detailed biological characterization of two non-CGD A. nidulans clinical isolates and compared the results to those with a reference strain. Phenotypic, metabolomic, and genomic analyses highlight fundamental differences in carbon source utilization, stress responses, and maintenance of cell wall integrity among the strains. One clinical strain had increased virulence in models with impaired neutrophil function. Just as in A. fumigatus, strain heterogeneity exists in A. nidulans clinical strains that can define virulence traits.

## Abstract (precis)

Immunocompromised patients are susceptible to infections with opportunistic filamentous fungi from the genus *Aspergillus*. Although A. fumigatus is the main etiological agent of *Aspergillus* species-related infections, other species, such as A. nidulans, are prevalent in a condition-specific manner. A. nidulans is a predominant infective agent in patients suffering from chronic granulomatous disease (CGD). A. nidulans isolates have mainly been studied in the context of CGD although infection with A. nidulans also occurs in non-CGD patients. This study carried out a detailed biological characterization of two non-CGD A. nidulans clinical isolates and compared the results to those with a reference strain. Phenotypic, metabolomic, and genomic analyses highlight fundamental differences in carbon source utilization, stress responses, and maintenance of cell wall integrity among the strains. One clinical strain had increased virulence in models with impaired neutrophil function. Just as in A. fumigatus, strain heterogeneity exists in A. nidulans clinical strains that can define virulence traits.

## INTRODUCTION

Fungal pathogen-related infections are now estimated to result in a higher number of human deaths than tuberculosis or malaria alone (1–3). The majority of systemic fungal infections are caused by *Candida* spp., *Pneumocystis* spp., *Cryptococcus* spp., and *Aspergillus* spp. (4, 5). Of the hundreds of known *Aspergillus* spp., only a few cause disease in animals, with the most prominent being Aspergillus fumigatus, Aspergillus flavus, Aspergillus nidulans, Aspergillus niger, and Aspergillus terreus (6, 7).

The primary route of infection of *Aspergillus* spp. is via the inhalation of conidia (asexual spores). In immunocompetent individuals, inhaled conidia are rapidly cleared by pulmonary resident and recruited neutrophils and macrophages, together preventing the onset of infection (8–10). However, disturbances to the immune system may render an individual susceptible to infection by *Aspergillus* spp. (11). The severity of infection largely depends on fungal species and genotype, the host immunological status, and host lung structure (6). Invasive aspergillosis (IA) is the most severe disease caused by *Aspergillus* spp. and is characterized by systemic host invasion, resulting in high mortality rates (30 to 95%) (2, 10, 11).

Patient populations with a highest risk of IA are (i) those with prolonged neutropenia from intensive myeloablative chemotherapy, (ii) cancer patients who are immunosuppressed due to chemotherapy and/or radiotherapy, (iii) those with cystic fibrosis, a hereditary disease that affects the lungs, (iv) and those with genetic disorders resulting in primary immune deficiencies, such as chronic granulomatous disease (CGD) (12, 13). CGD is a genetic disorder that affects 1 in 250,000 people, and in ∼80% of all cases subjects are of the male sex. CGD is caused by mutations in the genes encoding any of the five structural components of the NADPH-oxidase complex, an enzyme complex important for superoxide anion and downstream reactive oxygen species (ROS) production in phagocytic cells (14). As a result, immune cells are unable to efficiently kill microorganisms, and these microorganisms can then become pathogenic in such patients (13, 14)

Although A. fumigatus is the main etiological agent of *Aspergillus*-related infections in immunocompromised patients, other *Aspergillus* spp. have been found to have a high infection rate under some conditions. A. nidulans infections are not commonly reported in immunocompromised patients, except for subjects suffering from CGD (15, 16). In CGD patients, A. fumigatus and A. nidulans are responsible for 44% and 23%, respectively, of all fungal infections (15, 16). Infections with A. nidulans cause mortality in 27 to 32% of CGD patients (15), and in comparison to A. fumigatus, A. nidulans isolates have high virulence, invasiveness, dissemination, and resistance to antifungal drugs in these patients (17). Hence, A. nidulans infections have been studied mainly in the context of CGD although this fungal species can also be virulent in non-CGD, immunocompromised patients (18). In comparison to research on A. fumigatus, investigations into A. nidulans isolate virulence have been neglected, with very few studies having investigated the genetic and metabolic features of A. nidulans clinical strains, isolated from CGD and non-CGD patients, in the context of stress responses encountered during human host infection as well as during interactions with host immune responses (18–21).

The aim of this work was to carry out a detailed molecular, phenotypic, and virulence characterization of two A. nidulans clinical isolates (CIs) from (i) a patient with breast carcinoma and pneumonia and (ii) a patient with cystic fibrosis who underwent lung transplantation and to compare the results to those with the well-characterized, wild-type isolate FGSC A4 (A4).

## RESULTS

### A. nidulans clinical isolates have increased growth in comparison to that of the reference strain in the presence of alternative carbon sources.

**FIG 1:**
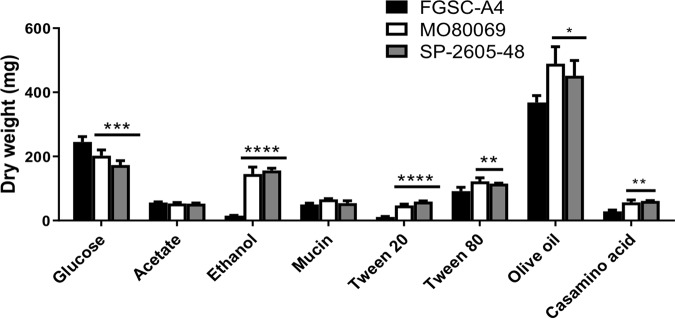
The A. nidulans clinical isolates exhibit improved growth in the presence of alternative carbon and lipid sources. Strains were grown in liquid MM supplemented with glucose, acetate, ethanol, mucin, Tween 20 and 80, olive oil, and Casamino Acids at 37°C for 48 h (glucose) or 72 h (others) before fungal biomass was freeze-dried and weighed. Standard deviations were determined from biological triplicates in a one-way ANOVA with Tukey’s posttest comparing growth of the clinical isolates to that of the FGSC A4 reference strain (*, *P* < 0.05; **, *P < *0.01; ***, *P < *0.001; ****, *P < *0.0001).

Fungal metabolic plasticity, which allows growth in unique and diverse ambient and host microenvironments, has long been hypothesized to contribute to *Aspergillus* virulence, with carbon sources such as glucose (22), ethanol (23), and acetate (24) being predicted to be actively used during *in vivo* infection. In addition, fatty acids and lipids are also thought to serve as major nutrient sources during mammalian host colonization, as is evident by the importance of key glyoxylate cycle enzymes in fungal virulence (25). We therefore characterized growth by determining the fungal dry weight of the two A. nidulans CIs in the presence of minimal medium (MM) supplemented with different physiologically relevant carbon sources, namely, glucose, acetate, ethanol, and lipids, and compared the results to those with the FGSC A4 reference strain. A significant reduction in growth was observed for both CIs in the presence of glucose, whereas both CIs had significantly increased growth in the presence of the alternative carbon sources ethanol, Casamino Acids, and the lipids Tween 20 (a source of lauric, palmitic, and myristic acids) (26), Tween 80 (which contains principally oleate) (26), and olive oil (triacylglycerols and free fatty acids) (27) (Fig. 1). In contrast, no difference in fungal biomass accumulation was observed in the presence of acetate and the lung-resident glycoprotein mucin (Fig. 1). These results suggest that the A. nidulans CIs have improved growth relative to that of the reference strain in the presence of most of the alternative carbon sources tested here, including different lipids.

### Metabolic profiles differ among the A. nidulans clinical isolates and the reference strain in the presence of different carbon sources.

To further investigate nutrient utilization in the A. nidulans CIs, the metabolic profiles of strains MO80069 and SP-2605-48 were determined and compared to the profile of the reference strain A4. Metabolomics was carried out on cellular extracts from strains grown for 24 h in fructose-rich MM and then transferred for 16 h to MM supplemented with glucose (CIs present reduced growth), ethanol (CIs had increased growth), acetate, and mucin (no difference in growth profiles). A total of 40 different metabolites were identified when strains were grown in the presence of glucose and ethanol, whereas 44 different metabolites were identified when strains were grown in the presence of acetate and mucin (see Table S1; all supplemental material is posted at https://doi.org/10.6084/m9.figshare.11973936). In a comparison of the metabolite quantities of strain MO80069 to those of the reference strain, 18 (45%), 22 (55%), 23 (52%), and 24 (55%) metabolite quantities were significantly (*P* value of <0.05) different from the quantities in the reference strain when strains were grown in glucose, ethanol, acetate, and mucin, respectively (Table 1; see also Table S1). In strain SP-2505-48, 15 (38%), 23 (58%), 30 (68%), and 14 (32%) metabolite quantities, which were normalized by fungal dry weight, were significantly (*P* value of <0.05) different from the quantities in the reference strain in the presence of glucose, ethanol, acetate, and mucin, respectively (Table 1; see also Table S1). Principal-component analysis (PCA) and hierarchical clustering analysis (HCA) of identified metabolite quantities showed that the CIs clustered apart from the reference strain and from each other in all tested carbon sources (see Fig. S1 and S2 at the URL mentioned above), indicating that they are metabolically different from the reference strain and from each other.

**TABLE 1:** Number and percentage of identified metabolite quantities that were significantly different in the A. nidulans clinical isolates^*a*^

Carbon source (*n*)^*b*^	No. (%) of differentially produced metabolites	
	MO80069 vs FGSC A4	SP-2605-48 vs FGSC A4
Glucose (40)	18 (45)	15 (38)
Ethanol (40)	22 (55)	23 (58)
Acetate (44)	23 (52)	30 (68)
Mucin (44)	24 (55)	14 (32)

a Metabolite quantities in A. nidulans clinical isolates were compared to those of the reference strain (*P* < 0.05). Strains were grown in the presence of the indicated carbon source for 16 h.

b *n*, number of metabolites tested.

**FIG 2:**
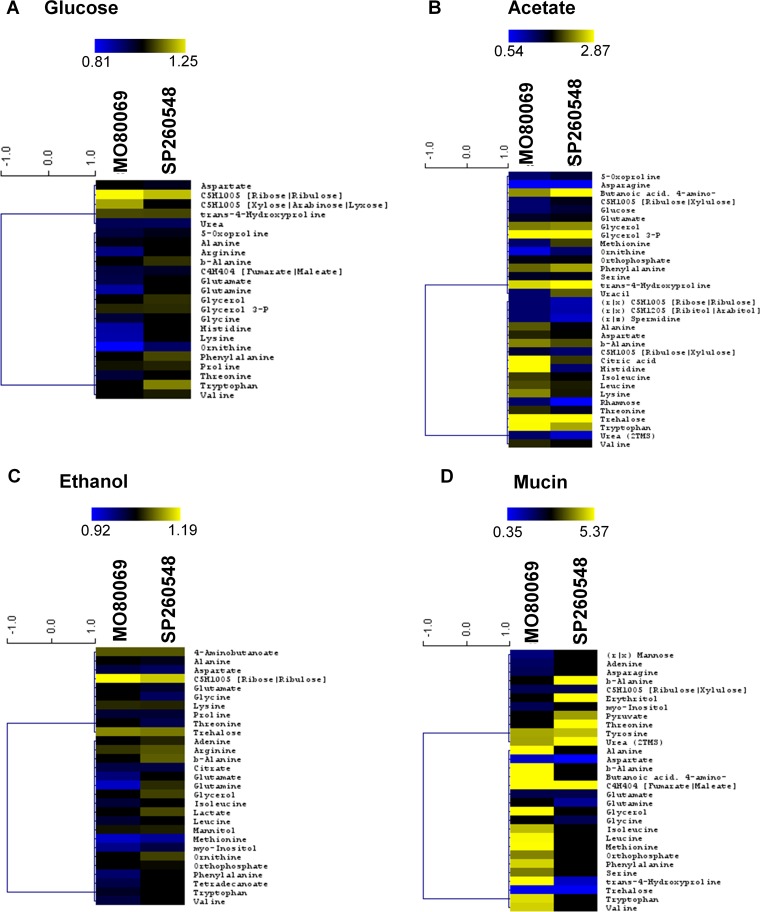
The A. nidulans clinical isolates are metabolically different from the reference strain in the presence of different carbon sources. (A to D) Heat maps depicting log fold changes of identified metabolite quantities that were significantly (*P < *0.05) different in the A. nidulans clinical isolates MO80069 and SP-2605-48 compared to levels in the FGSC A4 reference strain (gray squares depict metabolite quantities that were not detected as significantly different in one of the clinical isolates).

When we further focused on metabolites that were significantly different in quantity between the CIs and the reference strain, we observed that in the presence of glucose and ethanol, the majority of identified metabolites were present in significantly lower quantities than in the reference strain whereas both CIs had significantly higher metabolite quantities in the presence of acetate than the reference strain (Fig. 2A to C). Furthermore, when the A. nidulans CIs were cultivated in mucin-rich minimal medium, only 9 out of 29 significantly different metabolite quantities were identified in both strains whereas the remaining metabolite quantities were strain specific, suggesting that the metabolic profiles of the two differed drastically in the presence of this carbon source (Fig. 2D).

When the CIs were grown in a glucose-rich MM, amino acids were found in lower quantities in both CIs than in the reference strain. In contrast, pentose phosphate pathway (PPP) intermediates, glycerol, glycerol derivatives, and aromatic amino acids were detected in significantly higher quantities in this carbon source (Fig. 2A). In an ethanol-rich MM, significantly lower quantities of various amino acids as well as of the citric acid cycle intermediate citrate were detected in the CIs whereas increased quantities of several amino acid pathway intermediates, the carbon compounds glycerol, mannitol, and trehalose, PPP intermediates, and lactate were detected in the CIs compared to levels in the reference strain grown in this carbon source (Fig. 2C). In acetate-rich MM, most identified metabolites, notably a variety of amino acids, were present in significantly larger amounts in the CIs than in the reference strain, with the exception of some amino acids, PPP intermediates, spermidine, rhamnose, and urea (Fig. 2B). When strains were grown in mucin-rich MM, differences in the quantities of a variety of amino acids were observed, whereas trehalose was present in significantly lower quantities and urea was present in significantly higher quantities in both CIs than in the reference strain (Fig. 2D). In summary, these results suggest significant differences in amino acid biosynthesis and degradation, carbon source storage compounds, and degradation among the different A. nidulans strains in a condition-dependent manner.

To determine if any metabolic pathways were specifically enriched in the A. nidulans CIs in comparison to levels in the reference strain, pathway enrichment analyses were carried out on the metabolome data from glucose-, ethanol-, acetate-, and mucin-grown cultures. In all tested carbon sources, with the exception of mucin for isolate SP-2605-48, there was significant enrichment for aminoacyl-tRNA biosynthesis (Table 2). The pathway constituting the metabolism of arginine and proline was significantly enriched in both clinical isolates when they were grown in the presence of glucose and ethanol and in isolate SP-2605-48 when it was incubated in mucin-rich medium (Table 2). When acetate was used as the sole carbon and energy source, enrichment of the metabolism of these amino acids was not observed (Table 2). In addition, metabolites identified for strain SP-2605-48 in the presence of mucin and ethanol showed pathway enrichment in nitrogen metabolism (Table 2). In agreement with the aforementioned differences in amino acid quantities, these results suggest that the CIs exhibit differences in nitrogen metabolism in a carbon source-independent manner compared to that of the reference strain.

**TABLE 2:** Significant metabolic pathway enrichments

Carbon source	Enriched pathways in:	
	MO80069	SP-2605-48
Glucose	Aminoacyl-tRNA biosynthesis, arginine and proline metabolism	Aminoacyl-tRNA biosynthesis, arginine and proline metabolism
Acetate	Aminoacyl-tRNA biosynthesis; alanine, aspartate, and glutamate metabolism; cyanoamino acid metabolism; valine, leucine, and isoleucine metabolism; glycine, serine and threonine metabolism	Aminoacyl-tRNA biosynthesis, beta- alanine metabolism
Ethanol	Aminoacyl-tRNA biosynthesis, arginine and proline metabolism	Aminoacyl-tRNA biosynthesis, arginine and proline metabolism, nitrogen metabolism, alanine, aspartate, and glutamate metabolism

### The A. nidulans clinical isolates are more sensitive to hydrogen peroxide-induced oxidative stress and cell wall-perturbing agents than the reference strain.

Due to the significant metabolic differences observed between the CIs and the reference strain in the presence of physiologically relevant carbon sources and given that primary metabolism (carbon source utilization) has been shown to impact virulence factors in opportunistic pathogenic fungi (28, 29), we hypothesized that similar differences could be observed in the presence of physiologically relevant stress conditions. One such virulence factor is the fungal cell wall, which is crucial for protection, interaction with, and modulation or evasion of the host immune system (30). In addition, cell wall polysaccharide composition is dependent on carbon source primary metabolism (28, 29, 31).

**FIG 3:**
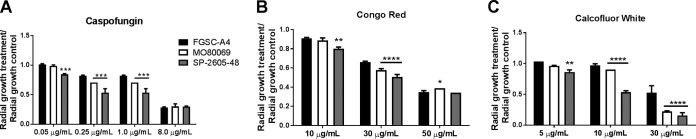
The A. nidulans clinical isolates are more sensitive to the cell wall-perturbing agents. (A to C) Strains were grown from 10^5^ spores on glucose minimal medium supplemented with increasing concentrations of caspofungin, Congo red, and calcofluor white for 5 days at 37°C. Standard deviations represent biological triplicates in a two-way ANOVA test, comparing growth of the clinical isolates to growth of the FGSC A4 reference strain (*, *P* < 0.05; **, *P* < 0.01; ***, *P* < 0.001; ****, *P* < 0.0001).

The production of reactive oxygen species (ROS), such as H_2_O_2_, and subsequent augmentation of cellular oxidative stress are strategies employed by the mammalian immune system to combat potential invading pathogenic microorganisms (14). The A. nidulans reference strain and the two CIs were therefore grown in the presence of hydrogen peroxide (H_2_O_2_) and the oxidative stress-inducing compound menadione. Both CIs were more sensitive (reduced growth) to high concentrations of H_2_O_2_ (see Fig. S3A at https://doi.org/10.6084/m9.figshare.11973936) whereas they were more resistant to menadione than the reference strain (see Fig. S3B). Furthermore, iron sequestration and elevated body temperature are additional physiological stress responses exerted by the host to prevent and/or control infection progression (32). Strains were therefore grown on iron-poor, glucose-rich minimal medium supplemented without (control) or with the iron chelators bathophenanthroline disulfonate (BPS) and ferrozine (see Fig. S3C), as well as in the presence of increasing temperatures (see Fig. S3D). Growth rates of all strains were similar under these conditions although strain MO80069 grew slightly more in the presence of the iron chelators (see Fig. S3C). Last, growth of all strains was assessed in the presence of the cell wall-perturbing agents caspofungin, Congo red (CR), and calcofluor white (CFW). The echinocandin caspofungin is a noncompetitive inhibitor of the cell wall enzyme β-1,3-glucan synthase (33) while CR and CFW bind to glucan and chitin chains, respectively (34, 35). CR and CFW therefore interfere with the cross-linking of cell wall polysaccharides, resulting in a reduction of cell wall stability. Both clinical isolates were more sensitive to low and medium concentrations of caspofungin than the reference strain, whereas all three strains grew similarly in the highest tested caspofungin concentration (8 μg/ml) (Fig. 3A). Similarly, both clinical strains were more sensitive to lower concentrations of CR whereas no significant difference in growth levels was observed in the presence of 50 μg/ml CR between all strains (Fig. 3B). In contrast, the CIs had significantly reduced growth in the presence of CFW compared to that of the reference strain (Fig. 3C).

In summary, the aforementioned results suggest strain-specific differences in the response to different physiological stress conditions and imply that the two A. nidulans CIs are more sensitive to cell wall-perturbing agents than the reference strain.

### The A. nidulans clinical isolates do not display increased resistance to azoles and amphotericin B.

Since both CIs showed increased susceptibility to caspofungin, an echinocandin that is being used as a second-line treatment for fungal infections (33), and to other cell wall-perturbing agents, we expanded our analyses to include two additional antifungal drugs classes. Specifically, we followed guidelines for the diagnosis and management of aspergillosis, which, in most cases, recommends treating aspergillosis with azoles and polyene drugs (11), both of which are known to interfere with the biosynthesis or physicochemical properties of fungal membrane sterols (10). Therefore, we determined the MICs of the azoles voriconazole and posaconazole and the polyene amphotericin B for all three strains. No differences in the MICs among all strains to these drugs was observed (Table 3).

**TABLE 3:** MICs of voriconazole, posaconazole, and amphotericin B for the A. nidulans clinical isolates MO80069 and SP-2605-48 and the FGSC A4 reference strain

Strain	MIC (μg/ml)		
	Voriconazole	Posaconazole	Amphotericin B
FGSC A4	0.25	1.0	2.0
MO80069	0.25	1.0	2.0
SP260548	0.25	1.0	2.0

### Cleistothecium formation is impaired in the A. nidulans SP-2605-48 strain.

A. nidulans is known for its easily inducible sexual cycle, which serves as a laboratory-based molecular tool for strain construction and studying fungal sexual reproduction (36). To further characterize A. nidulans CI biology, we performed self- and outcrosses for each clinical strain and the reference strain (control) at 30 and 37°C to assess whether A. nidulans CIs are able to undergo sexual reproduction.

Strains were first crossed with themselves (self-crosses) at 30°C and 37°C, and cleistothecium formation was observed for all strains at both temperatures, except for strain SP-2605-48 at 37°C (Table 4). Density of cleistothecia (number of cleistothecia/square centimeter) also varied between strains in a temperature-dependent manner, with the clinical isolates forming fewer cleistothecia per square centimeter than the reference strain at 30°C and 37°C (Table 4). In addition, no difference in levels of ascospore viability was observed among strains (Table 4).

**TABLE 4:** Cleistothecium formation and density and ascospore viability resulting from diverse A. nidulans self- and outcrosses

Temp (°C)	Cross^*a*^	Cleistothecium production	Cleistothecium density (no. of cleistothecia/cm2)	Ascospore viability (%)
30	A4 × A4	Yes	15.0 ± 0.81	91.83 ± 3.53
	MO × MO	Yes	7.0 ± 1.35	92.83 ± 3.96
	SP × SP	Yes	0.25 ± 0.25	89.83 ± 3.51
	MO × R21	Yes	1.25 ± 0.25	94.83 ± 3.85
	SP × R21	No		
37	A4 × A4	Yes	9.75 ± 1.43	90.67 ± 3.62
	MO × MO	Yes	5.25 ± 1.31	92.5 ± 2.76
	SP × SP	No		
	MO × R21	Yes	5.0 ± 0.40	92.5 ± 1.28
	SP × R21	No		

a A4, FGSC A4 reference strain; MO, MO80069 clinical isolate; SP, SP-2605-48 clinical isolate; R21, R21XR135, *paba*-deficient strain.

Outcrosses were performed by crossing the *pyrG* (requirement for uridine and uracil) auxotrophic strains MO80069 and SP-2605-48 with the *paba* (requirement for para-aminobenzoic acid)-deficient strain R21XR135 (Table 5). Strain MO80069 produced cleistothecia at both 30 and 37°C whereas strain SP-2605-48 did not produce any cleistothecia under any of the tested conditions. Density of cleistothecia was very low at 30°C (1.25 cleistothecia/cm^2^) but increased to the same number observed for the self-crosses at 37°C, with high ascospore viability in all cases (Table 4).

**TABLE 5:** Strains used in this study

Strain	Genotype	Source	Reference
FGSC-A4	Glasgow wild type (*veA^+^*)	Soil	36
MO80069	Wild type, clinical isolate	Bronchoalveolar lavage sample from a patient with breast carcinoma and pneumonia (Portugal)	This study
SP-2605-48	Wild type, clinical isolate	Patient with cystic fibrosis who underwent lung transplantation (Belgium)	This study
R21XR135	*pabaA1 yA2*	NA^*a*^	This study
MO80069 *pyrG*^−^	*pyrG89*	This study	This study
SP-2605-48 *pyrG^−^*	*pyrG89*	This study	This study
*ΔmpkA* strain	*ΔakuB mpkA*::*ptrA PTR*	NA	75

a NA, not applicable.

### Identification of SNPs and copy number variations in the A. nidulans clinical isolate genomes.

The aforementioned phenotyping and metabolomics results indicate differences between the strains that affect traits such as nutrient source utilization and resistance to different stresses. These results are in agreement with studies in A. fumigatus that have described great strain heterogeneity in traits such as growth, fitness, and enzyme secretion between different environmental and clinical isolates (24, 37). Indeed, the number of single nucleotide polymorphisms (SNPs), obtained during strain pairwise comparison, in the genomes of different A. fumigatus strains range between ∼13,500 (24) and ∼50,000 (38, 39). Strain heterogeneity has therefore mainly been investigated in environmental and clinical isolates of A. fumigatus, whereas similar studies have not been carried out for A. nidulans isolates. We therefore decided to determine differences at the genomic level by sequencing the genomes of our two A. nidulans CIs and comparing the sequences to the sequence of the FGSC A4 reference genome.

The genomes of MO80069 and SP-2605-48 aligned at 98.3% and 97.4%, respectively, to the genome of the reference strain FGSC A4, with 99.8% nucleotide identity. On the other hand, 1.5% and 1.9% of the A4 assembled genome did not align to the MO80069 and SP-2605-48 genomes, respectively, indicating differences among the genomes of all three strains.

**FIG 4:**
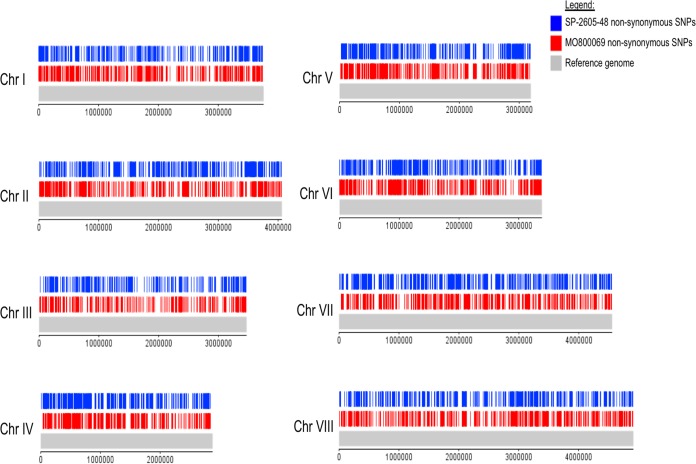
Diagram depicting the location of all detected nonsynonymous single nucleotide polymorphisms (SNPs) on the 8 chromosomes (Chr I to Chr VIII) of the A. nidulans clinical isolates SP-2605-48 and MO80069 in comparison to the FGSC A4 reference genome.

**FIG 5:**
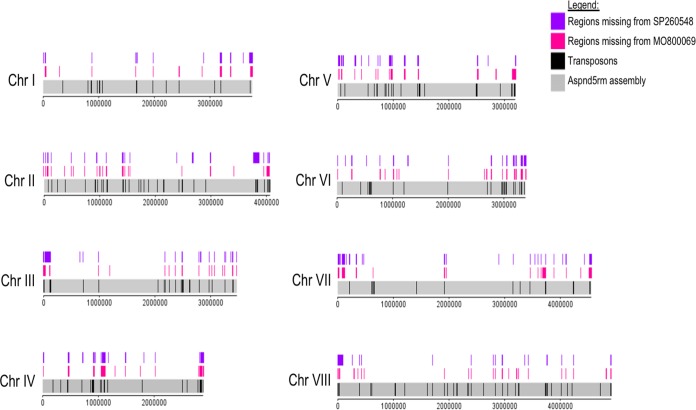
Diagram depicting the location of all detected small deletions on the 8 chromosomes (Chr I to Chr VIII) of the A. nidulans clinical isolates SP-2605-48 and MO80069 in comparison to the FGSC A4 reference genome. Also shown are the locations of putative transposons in the A. nidulans reference genome.

A total of 12,956 and 12,399 SNPs with respect to the A4 reference genome were detected in the genomes of MO80069 and SP-2605-48, respectively (Table 6; see also Table S2 at https://doi.org/10.6084/m9.figshare.11973936). When the genome of SP-260548 was compared to the genome of MO80069, 12,836 SNPs were detected (Table 6; see also Table S2). Each SNP mutation was classified as either high, moderate, or low according to its impact on the DNA codon frame and amino acid sequence. High-impact-type mutations encompass frameshift mutations and stop codon gain/loss, whereas missense mutations, resulting in amino acid changes, are considered moderate-impact-type mutations. Low-impact-type mutations contain all synonymous mutations and mutations within gene introns and untranslated regions (UTRs). The genome of MO80069 contained 501 high-impact mutations, 6,271 missense (moderate impact) mutations, and 6,184 synonymous (low impact) mutations in comparison to the sequence of the reference genome (Table 6; see also Table S2). In the genome of SP-2605-48, 465 high-impact mutations, 5,896 moderate-impact mutations, and 6,038 low-impact mutations were detected in comparison to the sequence of the reference genome (Table 6; see Table S2). When the genomes of both CIs were compared, 426 high-impact mutations, 6,288 missense mutations, and 6,122 synonymous mutations were detected (Table 6; see also Table S2 at the URL mentioned above). All nonsynonymous mutations were distributed throughout the genomes of both CIs, and no clear pattern in mutation accumulation could be observed for any of the 8 chromosomes (Fig. 4 and 5).

**TABLE 6:** Type and number of SNPs and long indels detected between the genomes of the A. nidulans clinical isolates MO80069 and SP-2605-48 and compared to the FGSC A4 reference genome

Mutation type	No. of mutations		
	MO80069 vs FGSC A4	SP-2605-48 vs FGSC A4	SP-2605-48 vs MO80069
SNPs			
Stop codon gain/loss	149	110	170
Frameshift	352	355	256
Missense	6,271	5,896	6,288
Synonymous	6,184	6,038	6,122
Total	12,956	12,399	12,836
Indels			
Insertions	234	308	222
Deletion	114	138	207
Total	348	446	375

In addition, the genomes of both CIs were screened for large-scale (>50 bp) insertions and deletions (indels). In total, 1,169 large-scale indels, consisting of anything between 3 bp to 23 kbp in size, were detected among the eight chromosomes of the CIs compared to the genome of the reference strain (see Table S3). Of these, 348 indels were specifically located in the genome of MO80069, 446 indels were found in the genome of SP-2605-48 only, and 375 indels were located in the genomes of both CIs (Table 6; see also Table S3). The majority of these indels were insertions (Table 6). Of the 375 indels found in the genomes of both CIs, 227 (60.5%) indels differed between the two strains, with the remaining 148 indels being identical for both strains (see Table S3 at the URL mentioned above).

### The A. nidulans clinical isolates are defective in MpkA accumulation in response to cell wall stress.

As this work aimed to characterize metabolic utilization of physiologically relevant carbon and lipid sources in A. nidulans CIs, including acetate and fatty acids, we screened genes encoding proteins important for carbohydrate and lipid utilization, cell wall biosynthesis/remodeling, and sexual reproduction for the presence of any of the aforementioned moderate- and high-impact mutations (see Table S4 at the URL mentioned above). Moderate-impact (missense) mutations were detected in three genes (*hxkA*, *swoM*, and *pfka*), encoding proteins involved in glycolysis (hexokinase, glucose-6-phosphate isomerase, and 6-phosphofructokinase) in both CIs, whereas four and six missense mutations were found in two genes (*idpA* and *mdhA*) encoding the enzymes isocitrate dehydrogenase and malate dehydrogenase of the tricarboxylic acid cycle in the genomes of MO80069 and SP-2605-48, respectively (see Table S4). Similarly, several moderate-impact mutations were found in genes encoding enzymes required for C_2_-associated metabolism (acetate, ethanol, and fatty acid), including *farA* (transcription factor regulating fatty acid utilization) and *farB* (transcription factor regulating the utilization of short-chain fatty acids) in both CIs, *facA* (acetyl-coenzyme A [CoA] synthase), *acuM* (transcriptional activator required for gluconeogenesis), and *alcM* (required for ethanol utilization) in SP-2605-48, and *echA* (enoyl-CoA hydratase) in MO80069 (see Table S4). Genes encoding proteins that function in the glyoxylate cycle also contained missense mutations in both CIs (see Table S4). Furthermore, a frameshift mutation was detected in both CIs in *acuL*, encoding a mitochondrial carrier involved in the utilization of carbon sources that are metabolized via the Krebs cycle (40) (see Table S4 at the URL mentioned above). The aforementioned mutations could underlie the observed differences in phenotypic growth in the presence of different carbon and lipid sources.

Due to the absence of cleistothecium formation in strain SP-2605-48, we wondered whether this strain contained any mutations in genes encoding proteins required for A. nidulans sexual reproduction. We found 11 and 13 mutations in 7 and 9 genes related to mating in the MO80069 and SP-2605-48 genomes, respectively (see Table S4). These mutations include missense and frameshift mutations in genes involved in the perception of light and dark (*ireA*, *ireB*, *cryA*, *veA*, and *velB*), mating processes (*cpcA*, *rosA*, and *nosA*), and signal transduction (*gprH* and *gprD*) (see Table S4). Indeed, *rosA* was absent in both CIs whereas *ireA* was missing from the genome of SP-2605-48. RosA is a transcriptional repressor of sexual development (41) whereas IreA is a transcription factor required for the blue light response, important for developmental processes, including mating.

Last, as both CIs were sensitive to cell wall-perturbing agents, we screened for mutations in genes encoding enzymes involved in cell wall biosynthesis and degradation. Compared to the FGSC A4 reference genome, we found 159 and 90 mutations in 40 and 34 genes involved in cell wall biosynthesis, integrity, and signaling in the genomes of MO80069 and SP-2605-48, respectively (see Table S4 at https://doi.org/10.6084/m9.figshare.11973936). The majority of these mutations were moderate-impact missense mutations in genes that encode components required for 1,3-β- and α-glucan and chitin synthesis and degradation, including various types of glucanases, chitinases, and chitin synthases (see Table S4). However, 17 (MO80069) and 9 (SP-2605-48) mutations were high-impact-level mutations which occurred in genes AN0550 (putative glucan 1,3-beta-glucosidase), AN0509 (putative chitinase), AN0517 (putative chitinase), AN0549 (putative chitinase), AN9042 (putative alpha-1,3-glucanase), AN6324 (putative α-amylase), AN4504 (putative endo-mannanase), and AN0383 (putative endo-mannanase) (see Table S4). In addition, small frameshift mutations were detected in three genes encoding the mitogen-activated protein kinase (MAPK) kinase BckA (AN4887), the MAPK MpkA (AN5666), and the transcription factor RlmA (AN2984) (see Table S4). In A. fumigatus, BckA and MpkA are components of the cell wall integrity (CWI) pathway, which ensures the integrity of the cell wall and is activated in response to different cell wall stresses, including those exerted by cell wall-targeting antifungal drugs (42). RlmA was shown to act downstream of MpkA, regulating cell wall biosynthesis-related genes, and this transcription factor is also involved in the direct regulation of MpkA (43). Mutation in *rlmA* was observed only in the genome of strain SP-2648-05.

**FIG 6:**
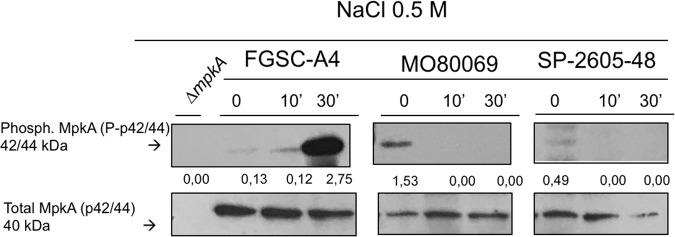
MpkA is not phosphorylated in the A. nidulans clinical isolates MO80069 and SP-2605-48 in the presence of NaCl-induced cell wall stress in contrast to MpkA levels in the FGSC A4 reference strain. Strains were grown from 1 × 10^7^ spores in complete medium for 16 h (control, 0 min) at 37°C before 0.5 M NaCl was added for 10 min (10′) and 30 min (30′). Total cellular protein was extracted, and Western blotting was carried out probing for phosphorylated MpkA. Signals were normalized by the amount of total MpkA present in the protein extracts, and cellular extracts from the Δ*mpkA* strain were used as a negative control.

In order to determine whether the observed frameshift mutations had an impact on CWI signaling, we carried out Western blotting of phosphorylated MpkA in the presence of NaCl-induced cell wall stress in all three A. nidulans strains. Phosphorylated MpkA levels were normalized by total cellular MpkA. Low levels of phosphorylated MpkA were detected in the absence of NaCl in all three strains, but whereas MpkA protein levels significantly increased upon cell wall stress in the FGSC A4 reference strain, no phosphorylated MpkA could be detected in either CI (Fig. 6). These results suggest that the observed frameshift mutations in *mpkA* had an effect on MpkA protein levels in the presence of cell wall stress, potentially being (one of) the cause(s) for the observed increased sensitivity to cell wall-perturbing agents.

### The A. nidulans clinical isolates do not display increased resistance to *in vitro*-mediated killing by different types of macrophages and neutrophils.

**FIG 7:**
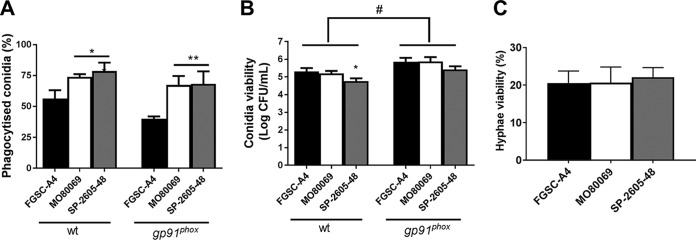
The A. nidulans clinical isolates MO80069 and SP-2605-48 do not present increased survival in the presence of macrophages and neutrophils. (A) Percentage of phagocytized conidia by murine wild-type and gp91^phox^ knockout macrophages. Macrophages were incubated for 1.5 h with conidia from the respective strains before phagocytized conidia were counted. (B) CFU counts as a measure of conidium viability after passage through wild-type (wt) and gp91^phox^ knockout macrophages. Macrophages were incubated with the respective conidia for 1.5 h before they were lysed, and contents were plated on complete medium. (C) Percentage of viable hyphal germlings after incubation for 16 h with neutrophils from healthy human donors. Strain viability was calculated relative to incubation without PMN cells, which was set at 100% for each sample. Standard deviations represent biological triplicates in a one-way ANOVA test with Tukey’s posttest (*, *P < *0.05; **, *P < *0.01, for results for the clinical isolates compared to those for FGSC A4; #, *P < *0.05, for a comparison of results for the two types of macrophages in the same strain).

Due to the observed phenotypic and genotypic differences, we wondered whether the CIs were different in virulence from the reference strain. Virulence was first characterized under a variety of *in vitro* conditions. Macrophages play an essential role in clearing *Aspergillus* species conidia from the lung (8), whereas neutrophils are predicted to primarily be responsible for eliminating fungal hyphae (39). To determine whether any strain-specific differences exist in macrophage-mediated phagocytosis and killing, the respective assays were carried out for all three strains in the presence of murine wild-type and gp91^phox^ knockout (CGD) macrophages. Macrophages from CGD patients are impaired in eliminating conidia from the lung environment, thus rendering the host more susceptible to fungal infections (20). Both types of macrophages phagocytosed a significantly higher number of conidia from both A. nidulans clinical isolates (∼75%) than the reference strain (∼50%) (Fig. 7A). Indeed, conidia from all three A. nidulans strains had increased levels of viability after phagocytosis by gp91^phox^ knockout macrophages than wild-type macrophages, confirming the inability of this type of macrophage to efficiently kill fungal conidia (Fig. 7B). Despite increased phagocytosis of both CIs, no difference in conidial viability levels was observed for strain MO80069 compared to the level of the reference strain, whereas wild-type but not CGD macrophages succeeded in killing significantly more SP-2605-48 conidia (Fig. 7B).

When challenged with human polymorphonuclear (PMN) cells, fungal survival was reduced approximately 80% for all three A. nidulans strains, indicating that the neutrophils were actively killing the hyphal germlings (Fig. 7C). No difference in strain survival rates was observed for the CIs (Fig. 7C). These results suggest that the A. nidulans CIs do not have higher survival rates in the presence of macrophages and neutrophils.

### Virulence of the A. nidulans clinical isolates depends on the host immune status.

**FIG 8:**
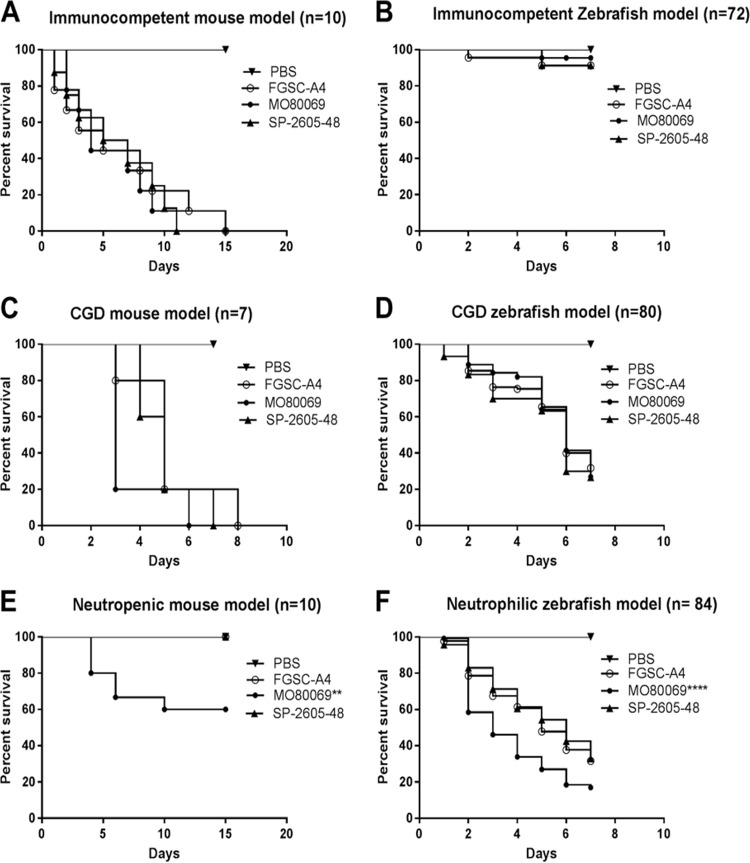
A. nidulans strain-specific virulence depends on the host immune status. The virulence of the A. nidulans clinical isolates MO80069 and SP-260548 was tested in murine (A, C, and E) and zebrafish (B, D, and F) models of pulmonary and invasive aspergillosis. Animals were manipulated in order to give rise to either immunocompetent (A and B), CGD (chronic granulomatous disease) (C and D), or neutropenic (E)/neutrophilic (F) models. Shown are survival curves for each immunosuppression condition and animal model. No difference in virulence levels was detected for all strains in both immunocompetent and CGD mice. Strain MO80069 was significantly more virulent in neutropenic mice and neutrophilic zebrafish. **, *P < *0.01; ****, *P < *0.0001 for a comparison of the values for the clinical isolates to those of the FGSC A4 reference strain in a two-way ANOVA test with Tukey’s posttest.

We determined the virulence of both A. nidulans CIs in animal models with different immune statuses. As it is well known that A. fumigatus strain-specific virulence is highly dependent on the type of host immunosuppression and model (24, 37, 43), we sought to determine if this would also be the case for A. nidulans. The virulence of A. nidulans CIs was assessed in both zebrafish and murine models of pulmonary and invasive aspergillosis. Furthermore, the immune system of each animal was manipulated in order to give rise to either immunocompetent, CGD, or neutropenic/neutrophilic models. As with patients, CGD models of both mice (19) and zebrafish (21) are very susceptible to A. nidulans infections. In both immunocompetent- and CGD-type zebrafish and mice, no difference in virulence levels between the A. nidulans clinical isolates and the reference strain was observed (Fig. 8A to D). However, the CI MO80069 was significantly more virulent in neutropenic mice and zebrafish with impaired neutrophil function than the reference strain, whereas no difference in virulence was observed for strain SP-2605-48 (Fig. 8E and F). These results suggest that, as in A. fumigatus, A. nidulans virulence depends on the strain and the host immune status.

## DISCUSSION

Aspergillus nidulans is a saprophytic fungus that can act as an opportunistic human pathogen in a host immune status- and genetic condition-dependent manner (15, 18, 44). Infection with A. nidulans is prevalent in patients with chronic granulomatous disease (CGD), and isolates have mainly been characterized in the context of this disorder (14, 15). Studies on A. nidulans virulence have been carried out in CGD models (animal and cell culture), and virulence characteristics have been compared to those of the primary human opportunistic fungus A. fumigatus (20, 21, 45, 46). A. fumigatus infection biology and characterization of strains that were isolated from immunocompromised patients under different conditions have received considerable attention in recent years (24, 37, 47), whereas similar studies into other pathogenic *Aspergillus* spp. have been neglected although it is becoming apparent that non-A. fumigatus species, including cryptic *Aspergillus* species, also contribute to host infection and invasion (7). This work therefore aimed at providing a detailed phenotypic, metabolic, genomic, and virulence characterization of two A. nidulans clinical isolates (CIs) that were isolated from non-CGD patients.

The first CI (MO80069) was isolated from a patient with breast carcinoma and pneumonia whereas the second CI (SP-2605-48) was obtained from a patient with cystic fibrosis who underwent lung transplantation. Genome sequencing confirmed these strains to be A. nidulans *sensu stricto*, and growth of these strains was characterized in the presence of physiologically relevant carbon sources. Fungi require carbon sources in large quantities in order to sustain biosynthetic processes and actively scavenge for them in their environment, including mammalian hosts (24). Available carbon sources vary according to the patient’s immune status and disease progression with, for example, corticosteroid treatment resulting in an increase of fatty and amino acid concentrations and a decrease of glucose levels in mouse lungs (22). Growth of the two A. nidulans strains in the presence of different carbon sources differed significantly from growth of the reference strain, with increased biomass accumulation being observed in the presence of alternative (ethanol, lipids, and amino acids) carbon sources and reduced growth in the presence of glucose. The observed phenotypic differences were corroborated by metabolic and genomic data which found a number of missense and high-impact mutations in genes encoding enzymes required for alternative carbon source and glucose utilization. These included missense mutations in genes encoding glycolysis- and citric acid cycle-related enzymes as well as five missense mutations in the transcription factor-encoding gene *farA*, which regulates the utilization of short- and long-chain fatty acids. Whether these mutations alone and/or in combination with other identified gene mutations are responsible for the observed growth phenotypes remains to be determined. Nevertheless, it is noteworthy that these mutations are found in both CIs, suggesting that these strains are able to grow well in nutrient-poor environments, such as the lung, compared to growth of the reference strain, which was isolated from the soil environment. Furthermore, whether these mutations are a result of adaptation to the host environment also remains subject to future investigations.

In addition, we also assessed the resistance of these strains to a variety of physiologically relevant stress conditions by growing them in the presence of oxidative stress and cell wall stress-inducing compounds, high temperature, iron limitation, and antifungal drugs. Some minor strain-specific differences were observed under these conditions, but the CIs were not significantly more resistant to these conditions, including exposure to azole- and polyene-type anti-fungal drugs, than the reference strain. It is possible that the patient-specific lung environment, biofilm formation, and/or interactions with other microorganisms may result in protection of these stresses, thus resulting in strains that do not have increased stress tolerance. In contrast to Candida albicans, an opportunistic fungal pathogen which was shown to interact with the Gram-negative bacterium Pseudomonas aeruginosa to promote colonization of patients with cystic fibrosis in a condition-dependent manner (48), such interactions have not been investigated for *Aspergillus* spp. *Aspergillus* interspecies interaction in lung microbiomes of patients with and without cystic fibrosis therefore remains an intriguing aspect of fungal pathobiology that warrants further characterization.

In contrast, both A. nidulans clinical strains were significantly more sensitive to the cell wall-perturbing agents calcofluor white, Congo red, and caspofungin (33–35) than the reference strain. These results suggest differences in cell wall composition and/or organization between the clinical isolates and the reference strain. When the respective genome sequences were analyzed, we found 159 and 90 mutations in 40 and 34 genes encoding enzymes required for cell wall glucan and chitin biosynthesis and degradation in strains MO80069 and SP-2605-48, respectively, compared to the genome of the FGSC A4 reference strain. Of particular interest was the identification of high-impact mutations in the genes *bckA*, *mpkA*, and *rlmA*, which encode components of the CWI signaling pathway. Indeed, Western blotting confirmed the absence of MpkA phosphorylation in the CIs in the presence of cell wall stress. These results suggest that the observed gene mutations cause an altered CWI response, resulting in increased sensitivity to cell wall-perturbing agents. The physiological relevance of these findings remains to be determined.

Aspergillus nidulans is characterized by an easily inducible sexual cycle as well as by undemanding laboratory-based cultivation and genetic manipulation conditions and has extensively been used as a model organism to study sexual reproduction and developmental processes (49). Nevertheless, it is unknown whether these traits can also be applied to A. nidulans clinical strains, and this work therefore assessed the ability of the two CIs to form cleistothecia in self- and outcrosses. Strain MO80069, similar to the reference strain, produced cleistothecia and viable ascospores under all tested conditions, whereas strain SP-2605-48 formed cleistothecia and viable ascospores only in self-crosses at 30°C and not at 37°C. This suggests that a certain degree of heterogeneity exists with regard to sexual reproduction in A. nidulans clinical strains although a bigger sample size and further studies are required in order to confirm this. Temperature has been shown to influence cleistothecium formation in *Aspergillus* spp., with lower temperatures of 30°C resulting in a higher number of formed cleistothecia (50). Furthermore, we cannot exclude the possibility that strains such as SP-2605-48 may require a different condition for sexual reproduction as it is determined by a series of environmental factors that can either activate or repress sexual development (50). This work identified six missense mutations in four genes (*veA*, *cpcA*, *fhbB*, and *gprH*) encoding enzymes involved in sexual development, and gene *ireA* was absent in the SP-2605-48 genome compared to the genomes of strains FGSC-A4 and MO80069. Genes *veA*, *cpcA*, *fhbB*, and *ireA* encode proteins that are involved in the perception of environmental signals (50), favoring the hypothesis that SP-2605-48 may require different/specific conditions for cleistothecium production, although it remains to be determined whether the aforementioned mutations and *ireA* are directly linked to the absence of cleistothecium production in strain SP-2605-48 under the conditions tested here.

Last, this work examined the *in vivo* virulence of the A. nidulans CIs in different animal models with a variety of immune statuses as A. fumigatus strain-specific virulence is highly dependent on the type of host immunosuppression and model (24, 37, 51). No difference in virulence levels was observed in immunocompetent and CGD murine and zebrafish models whereas strain MO80069 was significantly more virulent in a zebrafish with impaired neutrophil function and a neutropenic murine model of invasive aspergillosis than strains FGSC A4 and SP-2605-48. These results suggest that neutrophil recruitment and function at the site of infection are important for controlling A. nidulans infection in both vertebrates. Furthermore, results are in agreement with studies on A. fumigatus, which show that virulence is as much a strain-dependent as a host-dependent trait (24, 37, 39, 51). Furthermore, the tested phenotypes and genome mutations appear not to correlate with strain virulence although sample size has to be increased in order to confirm this in future studies. *Aspergillus* infection biology of mammalian hosts is a multifactorial and multifaceted process that depends not only on strain-specific virulence traits (30) but also on the genetic composition of the host and status of the immune system (52). Furthermore, the composition and interspecies interactions of the lung microbiome also influence pathogenicity of a given microorganism, with interactions between different species shown to influence host immune responses (49, 53). A. fumigatus is the main etiological agent of *Aspergillus*-related diseases and is predominantly present in the lung environment compared to the sites of infections caused by *Aspergillus* spp. (7). It is therefore possible that other *Aspergillus* spp., such as A. nidulans, remain largely undetected in the lung environment due to the predominant nature and/or inhibitory function of other fungal species and where they can grow without the necessity to evolve and adapt to extreme stress conditions. The prevalence and virulence of non-A. fumigatus species therefore remains a highly interesting and somewhat neglected topic that warrants future detailed studies. In summary, this is the first study that presents extensive phenotypic, metabolic, genomic, and virulence characterization of two A. nidulans clinical isolates. Just as in A. fumigatus, strain heterogeneity exists in A. nidulans clinical strains that can define virulence traits. Further studies are required to fully characterize A. nidulans strain virulence traits and pathogenicity.

## MATERIALS AND METHODS

### Ethics statement.

The principles that guide our studies are based on the Declaration of Animal Rights ratified by the UNESCO on the 27 January 1978 in its 8th and 14th articles. All protocols used in this study were approved by the local ethics committee for animal experiments from Universidade de São Paulo, Campus Ribeirão Preto (permit number 08.1.1277.53.6). All adult and larval zebrafish procedures were in full compliance with NIH guidelines and approved by the University of Wisconsin—Madison Institutional Animal Care and Use Committee (no. M01570-0-02-13).

### Strains, media, and growth conditions.

All strains used in this study are listed in Table 5. A. nidulans strain FGSC A4 was used as a reference strain. In addition to culture macroscopic features and fungal microscopic morphology analysis, whole-genome sequencing and phylogenetic analysis confirmed that both clinical isolates are A. nidulans (see Fig. S4; all supplemental material is available at https://doi.org/10.6084/m9.figshare.11973936). For phylogenetic tree construction, we compared *CaM*, *BenA*, *RPB2*, and *ITS* ribosomal DNA (rDNA) sequences, identified using blastN implemented in BLAST+, version 2.8.1 (54), to sequences from other species in the *Aspergillus* section *Nidulantes* (55), using a maximum-likelihood tree constructed with MEGA, version 10.1.1 (56). All strains were maintained in 10% glycerol at –80°C.

Strains were grown in either complete medium (CM) or minimal medium as described previously (57). Iron-poor MM was devoid of all iron and supplemented with 200 μM concentrations of the iron chelators bathophenanthrolinedisulfonic acid (4,7-diphenyl-1,10-phenanthrolinedisulfonic acid [BPS]) and 300 μM 3-(2-pyridyl)-5,6-bis(4-phenylsulfonic acid)-1,2,4-triazine (ferrozine). All growth was carried out at 37°C for the indicated amounts of time, except where otherwise stated (Fig. 1 and 3; see also Fig. S3). Reagents were obtained from Sigma-Aldrich (St. Louis, MO) except where otherwise stated. Radial growth was determined by inoculating plates with 10^5^ spores of each strain and incubation for 5 days before colony diameter was measured. Where required, the oxidative stress-inducing compound menadione or the cell wall-perturbing compounds Congo red (CR), caspofungin, and calcofluor white (CFW) were added in increasing concentrations. All radial growth was expressed as ratios, dividing colony radial diameter (in centimeters) of growth under the stress condition by colony radial diameter under the control (no stress) condition. To determine fungal dry weight, strains were grown from 3 × 10^6^ spores in 30 ml of liquid MM supplemented with 1% (wt/vol) glucose, acetate, mucin, or Casamino Acids or 1% (vol/vol) ethanol, Tween 20 and 80, or olive oil for 48 h (glucose) or 72 h (others) at 37°C and 150 rpm. All liquid and solid growth experiments were carried out in biological triplicates.

Growth in the presence of H_2_O_2_ was carried out as serial dilutions (10^5^ to 10^2^ spores) in liquid CM in 24-well plates for 48 h in the presence of different concentrations of H_2_O_2_.

### Metabolite analysis.

Metabolome analysis was performed as described previously (58). Briefly, metabolites were extracted from 5 mg of dry-frozen, mycelial powder of four biological replicates. The polar phase was dried, and the derivatized sample was analyzed on a Combi-PAL autosampler (Agilent Technologies GmbH, Waldbronn, Germany) coupled to an Agilent 7890 gas chromatograph coupled to a Leco Pegasus 2 time-of-flight mass spectrometer (LECO, St. Joseph, MI). Chromatograms were exported from the Leco ChromaTOF software, version 3.25, to the R software package (www.r-project.org). The Target Search R package was used for peak detection, retention time alignment, and library matching.

Metabolites were quantified by the peak intensity of a selective mass and normalized by dividing the value by the respective sample dry weight. Principal-component analysis was performed using the pcaMethods bioconductor package (59, 60). Pathway enrichment analysis was carried out using MetaboAnalyst (https://www.metaboanalyst.ca/faces/ModuleView.xhtml) (61).

### Determination of MICs.

MICs of amphotericin B, voriconazole, and posaconazole were determined by growing 10^4^ spores/well in 96-well plates containing 200 μl/well of RPMI medium and increasing concentrations of the aforementioned compounds, according to the protocol elaborated by the Clinical and Laboratory Standards Institute (62).

### Induction of cleistothecium formation.

Cleistothecium formation through self-crossing was induced by growing the strains on glucose minimal medium (GMM) plates that were sealed airtight and incubated for 14 days at 30 or 37°C. Plates were scanned for the presence of cleistothecia under a light microscope. To assess ascospore viability, five cleistothecia of each strain were collected, cleaned on 4% (wt/vol) agar plates, and resuspended in 100 μl of water. Ascospores were counted, and 100 ascospores were plated on GMM before CFU counts were determined. Cleistothecium density was determined through counting the number of cleistothecia of a certain area and dividing the value by the area (in square centimeters).

Cleistothecium formation through outcrossing was carried out as described previously (57). To induce *pyrG*^−^ auxotrophy in strains MO80069 and SP-2605-48 (Table 1), they were grown on GMM plates supplemented with 1.2 g/liter uridine and uracil (UU) and 0.75 mg/ml 5-fluoroorotic acid (FOA) in the form of a cross until single colonies appeared. Auxotrophy was confirmed by growing strains on GMM with and without UU before strains were crossed with strain R21XR135 (Table 1).

### DNA extraction, genome sequencing, and detection of SNPs and indels.

DNA was extracted as described previously (57). Genomes were sequenced using 150-bp Illumina paired-end sequence reads at the Genomic Services Lab of Hudson Alpha (Huntsville, AL). Genomic libraries were constructed with the Illumina TruSeq library kit and sequenced on an Illumina HiSeq 2500 sequencer. Samples were sequenced at greater than 180× coverage or depth.

The Illumina reads were processed with the BBDuk and Tadpole programs of BBMap release 37.34 (https://sourceforge.net/projects/bbmap/files/BBMap_37.34.tar.gz/download) to remove sequencing adapters and phiX and to correct read errors.

Two different Illumina assemblies were performed with the trimmed reads, using platanus (63) and sparseAssembler (64). Nanopore reads were first filtered for quality using Nanofilt (quality of >7) and then were corrected using Canu (65). Once corrected, a subset of reads covering 30 times the estimated genome size of 30 Mb was selected, giving preference to the longest reads. DBG2OLC (66) was used with each of the two Illumina assemblies and the subset of nanopore reads to perform two hybrid assemblies. Independently, MaSuRCA (67) was used to perform a hybrid assembly using the raw nanopore and Illumina reads. The three hybrid assemblies were then corrected using Pilon (68) for three rounds on each assembly. Ragout (69) was then used to fuse the three assemblies into one final assembly using the assembly obtained with MaSuRCA as the base and the other two as references. This assembly was then corrected again for three rounds using Pilon. The mitochondrial genome was obtained from the discarded contigs of MaSuRCA.

The Aspergillus nidulans FGSC A4 genome sequence and gene predictions, were obtained from the Aspergillus Genome Database (version s10-m04-r15 [http://aspgd.org/]). The processed DNA reads were mapped to the FGSC A4 genome with minimap2, version 2.17 (https://github.com/lh3/minimap2), and variants from the FGSC A4 sequence were called with Pilon, version 1.23 (https://github.com/broadinstitute/pilon). Short indels and nucleotide polymorphisms were recovered from the Pilon VCF files by filtering with vcffilter (https://github.com/vcflib/vcflib) to retain only calls with read coverage deeper than 7, exactly one alternative allele, and an alternative allele fraction of at least 0.8. Longer indels and sequence polymorphisms were recovered by searching the VCF files for the SVTYPE keyword. Support for the detected indels was verified by mapping reads to a modified version of the reference genome generated by Pilon. The read coverage depths over inserted sequences were compared to the coverage of flanking sequences, and deletion sites were checked for breaks in read coverage. Sequence variations inside predicted genes and their effects on predicted protein sequence were identified with a custom Python script. The mitochondrial genome was obtained from the discarded contigs of MaSuRCA. Due to its circular nature, the mitochondrial genome appeared repeated multiple times in a single contig. Lastal (http://last.cbrc.jp/doc/last.html) was used to extract one single copy of the mitochondrial genome using the reference mitochondrion.

### Detection of large genome deletions and insertions.

Genome assemblies of the two clinical isolates were aligned to the FGSC A4 reference genome with nucmer (70). The alignments were filtered to keep only one-to-one matches. Strain-specific loci were detected by searching the alignment coordinates table for regions of the A4 genome with no match in the clinical isolate genome. Large insertions were detected by searching the alignment coordinate table for regions of the clinical isolate genomes with no match in the A4 genome.

### Identification of transposon-like regions in the FGSC A4 reference genome.

Transposon-like regions were identified by running Pfam (71) on the six translation frames of the complete genome sequence. Regions containing any of the 14 domains typically known to be associated with transposable elements (see Table S1 at the URL mentioned above) were collected. Inverted repeats longer than 50 bp and separated by less than 5,000 bp were extracted and marked as potential miniature inverted-repeat transposable elements (MITE). The Pfam and MITE locations were combined to form the transposon track.

### Figure generation.

DNAPlotter (72) was used to display the loci of all nonsynonymous SNPs and large deletions identified in the two clinical strains compared to the reference genome of FGSC A4. In addition, the locations of transposon-like regions in the A4 genome were also highlighted using DNAPlotter.

### Western blotting.

Strains were grown from 1 × 10^7^ spores at 37°C and 200 rpm in 50 ml of CM for 16 h before being exposed to 0.5 M NaCl for 0, 10, and 30 min. Total cellular proteins were extracted according to Fortwendel and colleagues (73) and quantified according to Hartree (74).

For each sample, 60 μg of total intracellular protein was run on a 12% (wt/vol) SDS-PAGE gel before being transferred to a polyvinylidene difluoride (PVDF) membrane (GE Healthcare). Phosphorylated MpkA or total MpkA was probed for by incubating the membrane with a 1:5,000 dilution of the anti-phospho-p44/42 MAPK (9101; Cell Signaling Technologies) antibody or with a 1:5,000 dilution of the p44-42 MAPK (Cell Signaling Technology) antibody overnight at 4°C, with shaking. Subsequently, membranes were washed three times with TBS-T (2.423 g/liter Tris, 8 g/liter NaCl, 1 ml/liter Tween 20) and incubated with a 1:5,000 dilution of an anti-rabbit IgG horseradish peroxidase (HRP) antibody (7074; Cell Signaling Technologies) for 1 h at room temperature. MpkA was detected by chemiluminescence using a Western ECL Prime (GE Healthcare) blot detection kit according to the manufacturer’s instructions. Films were submitted to densitometric analysis using ImageJ software (http://rsbweb.nih.gov/ij/index.html). The amount of phosphorylated MpkA was normalized by the amount of total MpkA. The A. fumigatus Δ*mpka* strain was used as a negative control (Table 1) (75).

### Isolation and differentiation of BMDM.

Bone marrow-derived macrophages (BMDMs) were isolated as described previously (76). Briefly, BMDMs were recovered from femurs of C57BL/6 wild-type and gp91^phox^ knockout mice and were incubated in BMDM medium (RPMI medium [Gibco] supplemented with 30% [vol/vol] L929 growth-conditioning medium, 20% inactivated fetal bovine serum [FBS; Gibco], 2 mM glutamine, and 100 units/ml of penicillin-streptomycin [Life Technologies]). After 4 days, fresh medium was added for an additional 3 days before BMDMs were collected.

### *In vitro* phagocytosis and killing assays.

Phagocytosis and killing assays of A. nidulans conidia by wild-type and gp91^phox^ knockout macrophages were carried out according to Bom et al. (77) with modifications. Twenty-four-well plates containing a 15-mm-diameter coverslip in each well (phagocytosis assay) or without any coverslip (killing assay) and 2 × 10^5^ macrophages per well were incubated in 1 ml of RPMI-FBS medium (RPMI medium [Gibco] supplemented with 10% inactivated FBS[Gibco], 2 mM glutamine, and 100 units/ml of penicillin-streptomycin [Life Technologies]) at 37°C in 5% CO_2_ for 24 h. Wells were washed with 1 ml of phosphate-buffered saline (PBS) before the same volume of RPMI-FBS medium supplemented with 1 × 10^6^ conidia (1:5 macrophage/conidium ratio) was added under the same conditions.

To determine phagocytosis, macrophages were incubated with conidia for 1.5 h before the supernatant was removed, and 500 μl of PBS containing 3.7% formaldehyde was added for 15 min at room temperature (RT). Sample coverslips were washed with 1 ml of ultrapure water and incubated for 20 min with 500 μl of 0.1 mg/ml calcofluor white (CFW) to stain for the cell wall of nonphagocytized conidia. Samples were washed, and coverslips were viewed under a Zeiss Observer Z1 fluorescence microscope. In total, 100 conidia were counted per sample, and the phagocytosis index was calculated. Experiments were performed in biological triplicates.

To determine macrophage-induced killing of conidia, macrophages were incubated with conidia for 1.5 h before cell culture supernatants were collected and cytokine concentrations were determined. Macrophages were then washed twice with PBS to remove all nonadherent cells and subsequently lysed with 250 μl of 3% (vol/vol) Triton X-100 for 10 min at RT. Serial dilutions of lysed samples were performed in sterile PBS and plated onto CM and incubated at 37°C for 2 days before CFU counts were determined.

### PMN cell isolation and spore germination assay.

Human polymorphonuclear (PMN) cells from fresh venous blood of healthy adult volunteers were isolated according to Drewniak et al. (78), with modifications. Cells were harvested by centrifugation in isotonic Percoll, lysed, and resuspended in HEPES-buffered saline solution. A. nidulans asexual spores were incubated with PMN cells (1 × 10^5^cells/ml; ratio of 500 PMN:1 conidium) in a 96-well plate overnight at 37°C in RPMI 1640 medium containing glutamine and 10% fetal calf serum (Life). PMN cells were lysed in a solution of water and sodium hydroxide (pH 11.0) (Sigma-Aldrich), and spore germination was determined using an MTT (thiazolyl blue; Sigma-Aldrich) assay. Strain viability was calculated relative to incubation without PMN cells, the level of which was set at 100% for each sample. The viability of A. nidulans germinated spores in the presence of PMN cells was determined as described previously (32).

### *In vivo* infections in immunocompetent, CGD, and neutrophilic zebrafish.

We evaluated strain virulence in an established zebrafish-aspergillosis model. Seventy-two wild-type larvae were used as an immunocompetent model. Larvae with a dominant negative Rac2D57N mutation in neutrophils (*mpx*::*rac2D57N*) (39) were used as a model of leukocyte adhesion deficiency, where neutrophils do not reach the site of infection, and p22^phox^-deficient larvae [p22^phox (^*^sa11798^*^)^] were used as a chronic granulomatous disease (CGD) model (21).

Spore preparation and conidium microinjection into the hindbrain of 2-day postfertilization (dpf) larvae were performed as previously described (79). Briefly, after manual dechorionation of embryos, 3 nl of inoculum or PBS-control was injected into the hindbrain ventricle via the optic vesicle (∼50 conidia) in anesthetized larvae at approximately 36 h postfertilization.

### *In vivo* infections in immunocompetent, CGD, and neutropenic mice.

Virulence of the A. nidulans strains was determined in immunocompetent, CGD, and neutropenic mice. A. nidulans conidial suspensions were prepared, and viability experiments were carried out as described previously (77). Eight- to 12-week-old wild-type (*n* = 10) and gp91^phox^ knockout (*n* = 7) C57BL/6 male mice were used as immunocompetent and CGD models, respectively. Neutropenia was induced in 7- to 8-week-old BALB/c female mice (*n* = 10, weighing between 20 and 22 g) with cyclophosphamide at a concentration of 150 mg per kg, administered intraperitoneally (i.p.) on days −4 and −1 prior to infection (day 0) and at 2 days postinfection. Hydrocortisone acetate (200 mg/kg) was injected subcutaneously on day −3 prior to infection.

Mice were anesthetized and submitted to intratracheal (i.t.) infection as previously described (80) with some minor modifications. Briefly, after i.p. injection of ketamine and xylazine, animals were infected with 5.0 × 10^7^ (immunocompetent) or 1 × 10^6^ (CGD) conidia contained in 75 μl of PBS (81) by surgical i.t. inoculation, which allowed dispensing of the fungal conidia directly into the lungs. Neutropenic mice were infected by intranasal instillation of 1.0 × 10^4^ conidia as described previously (70). Phosphate-buffered saline (PBS) was administered as a negative control for each murine model.

Mice were weighed every 24 h from the day of infection and visually inspected twice daily. The endpoint for survival experimentation was identified when a 20% reduction in body weight was recorded, at which time the mice were sacrificed.

### Statistical analyses.

All statistical analyses were performed using GraphPad Prism, version 7.00 (GraphPad Software, San Diego, CA), with a *P* value of < 0.05 considered significantly different. A two-way analysis of variance (ANOVA) was carried out on all stress response tests whereas a one-way ANOVA with Tukey’s posttest was applied for growth in the presence of different carbon sources, for the phagocytosis index, and for the PMN cell killing assay. Survival curves were plotted by Kaplan-Meier analysis, and results were analyzed using a log rank test. All experiments were repeated at least twice.

### Data availability.

Short-read sequences for these strains are available in the NCBI Sequence Read Archive (SRA) under accession numbers SRR10983230, SRR10983231, SRR10983232, and SRR10983233 and BioProject number PRJNA603646. Genomes were deposited in GenBank under accession numbers JAAFYM000000000 and JAAFYL000000000.
